# Balancing work and life: how employee welfare programs influence mental health with a gendered lens

**DOI:** 10.1186/s40359-026-05235-5

**Published:** 2026-07-24

**Authors:** Alessia Valmori, Roberta Adorni, Claudia Manzi

**Affiliations:** 1 Catholic University of the Sacred Heart, Milan, Italy; 2 University of Milano-Bicocca, Milan, Italy

**Keywords:** Mental health, Gender differences, Work-life balance, Employee welfare

## Abstract

**Background:**

Balancing work and private life is challenging, particularly for women, who continue to shoulder more domestic responsibilities than men. This disproportionate burden, exacerbated during and after the pandemic, negatively impacts women’s mental health. While company welfare programs could help alleviate this imbalance, there has been limited research on their effectiveness.

**Methods:**

In our cross-sectional study (*N* = 7180 employees from four Italian companies, data were collected between September 2020 and July 2024), we investigated the role of employee welfare in reducing work-life conflict and enhancing mental health for both men and women. We tested a moderated mediation model in which satisfaction with employee welfare was linked to workers’ mental health through a reduction in perceived work-life conflict.

**Results:**

Descriptive analyses revealed that women more frequently reported lower levels of mental health compared to men. The mediational hypothesis was confirmed: satisfaction with welfare was associated with lower work-to-life conflict and with lower life-to-work conflict, leading to improved mental health for both genders. Gender moderated the relationship between employee welfare and mental health through work-to-life conflict, with the employee welfare being less supportive in reducing work-to-life conflict and in turn enhancing mental health for women.

**Conclusions:**

Overall, the results suggest that although welfare programs help reduce work-to-life conflict, they appear to be less effective in addressing life-to-work conflict and this is particularly detrimental for women.

**Supplementary Information:**

The online version contains supplementary material available at https://doi.org/10.1186/s40359-026-05235-5.

## Introduction

A persistent gender gap in mental health has emerged as one of the most documented inequalities in occupational health research, with women consistently reporting poorer mental well-being than men [1]. This disparity is deeply intertwined with the structural imbalances that characterize the intersection of work and private life: women continue to bear a disproportionate share of domestic and caregiving responsibilities, a burden that translates into higher levels of work-life conflict and, ultimately, reduced mental health.

In response to these challenges, many organizations have increasingly invested in employee welfare programs designed to help employees in balancing their work and private lives, with the aim of improving job satisfaction [2] and, in turn, enhancing company performance [3]. For example, in Italy some companies have focused on specific initiatives such as providing in-house childcare facilities for young children, allowing fully remote workdays, and partnering with nurseries and summer camps for employees’ children [4]. Despite these initiatives, the literature on the effectiveness of employee welfare remains limited, particularly regarding how these programs affect mental health across genders. Although women have increasingly entered the workforce and moved away from traditional roles of homemaking and childcare [5], societal structures have not evolved to fully compensate for these shifts. Consequently, women still bear a disproportionate share of household responsibilities [6] and experience higher levels of work-life conflict than their male counterparts [7, 8]. This conflict not only reduces job and family satisfaction but also contributes to lower overall well-being [1].

In this paper, we analyse a large dataset of Italian employees to examine how employee welfare programs influence mental health differently for men and women. We also explore the mediating role of perceived work-life conflict in this relationship. Building on previous research, we distinguish between two types of work-life conflict: work interfering with personal life and personal life interfering with work [9], investigating whether these conflicts are experienced differently by gender.

### Work-life conflict and the gender gap in mental health

According to the World Health Organization [10], mental health is defined as “a state of well-being in which the individual realizes his or her own abilities, can cope with the normal stresses of life, can work productively and fruitfully, and is able to make a contribution to his or her community”. To assess mental health, the WHO developed the 5-item World Health Organization Well-Being Index (WHO-5) [11], which has been translated into over 30 languages. The WHO-5 has proven crucial in screening for various clinical conditions such as depression and substance use disorders [12] and is widely used to measure general mental health across populations.

In this regard, numerous studies have documented the impact of the COVID-19 pandemic on men and women’s mental health globally. In line with Role Strain Theory [13], which posits that individuals have limited time and energy to meet the often competing expectations of multiple social roles, and Social Role Theory [14], which emphasizes how culturally prescribed norms assign women primary responsibility for caregiving and domestic work, the sudden intensification of both paid work and unpaid care during the pandemic has disproportionately increased women’s exposure to work–life conflict, thereby exacerbating their risk of stress, burnout, and poorer mental health outcomes. A recent review highlighted that gender disparities in mental health have been exacerbated, especially for working women and single parents. This is largely due to women’s disproportionate responsibility for domestic tasks, including childcare and eldercare [15]. Despite the workforce becoming more gender-balanced [5], with women increasingly taking on roles beyond traditional homemaking, societal structures have not kept pace. As a result, women in heterosexual couples still bear a larger share of household responsibilities, even when working full-time [6]. This ongoing imbalance places considerable strain on women’s mental health, with research consistently showing that women report lower levels of mental health compared to men [16], particularly in areas such as cheerfulness, calmness, and levels of depression and loneliness [17].

Thus, work-life conflict is widely recognized as a contributor to mental health inequalities [18], including the persistent gender gap in well-being [17]. The heavier multitasking burden often borne by women [19] limits their opportunities for stress recovery [20]. Recent research emphasizes the importance of examining work-life conflict from multiple perspectives. Not only work could interfere with personal life, but personal life could also disrupt work, and both can negatively affect mental health [9]. Previous studies indicate that work-to-life and life-to-work conflicts are distinct yet interrelated forms of inter-role conflict. Work-to-life conflict is typically driven by work-related stressors, such as long work hours, while life-to-work conflict is more often triggered by family-related stressors, like caring for young children [21]. Both types of conflict are linked to an increased risk of burnout [22] and serious physical and mental health issues, including back pain, headaches, sleep disorders, and depression [23].

Importantly, research suggests that women experience higher levels of life-to-work conflict compared to men, though no gender differences are found in the work-to-life direction [24]. This distinction highlights the complex nature of work-life balance challenges for women, as their family responsibilities often spill over into their professional lives, further compounding their mental health struggles.

This evidence acquires greater relevance in the Italian context. In Italy, unpaid domestic and care work falls disproportionately on women: women aged 20–74 spend 5 h and 9 min per day on unpaid work, compared with 2 h and 16 min for men [25]. Among women in full-time employment with children, the weekly gap in total workload (paid work + domestic tasks + childcare) reaches approximately 13 h compared to their partners [26, 27]. This pattern is consistent with European evidence that women are more likely to provide unpaid care and juggle multiple caring responsibilities [28]. In Italy, 85% of unpaid care work is carried out by women, and only 18% receive help from their partner [29].

This unequal distribution has direct implications for women’s mental health. National statistics [30] report that women’s mental health index is consistently lower than men’s across all age groups (66.5 vs. 70.9). Mental health services data show that women represent 55.9% of patients, with depression rates nearly double (46.5 vs. 27.0 cases per 10,000 inhabitants) [30]. Research indicates that unpaid domestic work is associated with poorer mental health specifically for women, with each hour associated with disproportionately greater mental health costs for women [31].

In this context, external support can be fundamental to help women in reducing the work-life conflict. For instance, organizational support could play a crucial role in addressing the gendered nature of work-life conflict, particularly in workplaces where the demands of professional and private life intersect most acutely.

### Addressing work-life conflict through employee welfare programs: a moderated mediational model

One form of organizational support is employee welfare programs, which have been shown to improve individuals’ perceptions of work-life balance [32]. Employee welfare encompasses employer initiatives aimed at supporting employees’ quality of life beyond wages and direct compensation [33]. These initiatives can span various areas of an employee’s life, including social, family, economic, and educational support, and overlap with the broader literature on work–family support policies and family-supportive organizational practices [34].

Welfare programs are mutually beneficial for both organizations and employees. From an organizational perspective, research [3] highlights a positive relationship between employee-centric policies and improved company performance. In addition, companies that prioritize employee welfare are often viewed as more socially responsible, a reputation that is becoming increasingly valuable in today’s socially conscious market [35]. For employees, welfare programs have been linked to higher job satisfaction [2] and improved perceptions of work-life balance [32], providing support in balancing personal and professional responsibilities.

Beyond traditional welfare provisions, a substantial literature has examined whether flexible work arrangements and work–family support policies help employees manage competing role demands. Meta-analytic evidence indicates that both the availability and use of work–family support policies are modestly associated with favourable employee outcomes, including lower work-to-family conflict and higher job satisfaction and affective commitment, particularly when employees perceive their organization as family-supportive [34]. A meta-analysis of flexible work arrangements likewise found that flexitime and flexplace can reduce work–family conflict, although effects are generally small and depend on the type of flexibility and the direction of conflict considered [36]. Recent evidence further suggests that flexible working can enhance employees’ quality of work life, although individual differences such as locus of control shape how employees experience and benefit from such arrangements [37]. Systematic review evidence also suggests that employee-oriented flexible work may yield modest benefits for mental health, especially when workers gain greater control over their schedules, although findings remain heterogeneous across study designs [38].

However, organizational support is not uniformly beneficial: its effectiveness depends on how policies are implemented and on employees’ job resources. Job autonomy and personal resources such as self-efficacy can jointly determine whether role stress undermines well-being in flexible or multi-role work contexts [39]. During the COVID-19 pandemic, mandatory remote work combined with after-hours connectivity demands intensified employees’ work–life tensions, highlighting how organizational and supervisory practices can amplify rather than alleviate conflict [40]. Forced teleworking has also been linked to counterproductive work behaviour through heightened work–family conflict, illustrating the need to distinguish between voluntary, well-designed flexibility and imposed arrangements that erode boundaries between work and private life [41]. Within this broader evidence base, organizational support may in some circumstances be beneficial for reducing work-life conflict. In a study of private and public organizations in Ireland [42], perceived support from immediate supervisors and HR managers influenced adherence to work–life balance initiatives, which in turn reduced employees’ overall work–life and inter-role conflict. Similarly, Batt and Valcour [43] reported that HR practices significantly predict lower work–life conflict and, as a consequence, lower turnover intention.

However, there is a notable gap in research regarding how employee welfare affects perceptions of work–life balance and mental health when considering gender. While previous studies have examined gender differences in work–life conflict and the effects of flexible work arrangements or organizational support on well-being, they have rarely investigated whether and how satisfaction with employee welfare programs translates into better mental health through reduced work-to-life and life-to-work conflict, and whether these processes operate differently for men and women. As women generally report lower levels of mental health [16], due to higher work–life conflict [7], it is therefore expected that organizational support targeted at reducing work–life conflict [42], such as employee welfare, may be especially beneficial for them.

Drawing on Role Strain Theory [13] and Social Role Theory [14], this study aims to address this gap by investigating the impact of employee welfare on men’s and women’s mental health, specifically through the lens of reducing work-to-life and life-to-work conflict. We test how satisfaction with employee welfare positively impacts employees’ mental well-being, by considering the mediational effect of work-to-life and life-to-work conflict. Importantly, we also consider the moderating effect of gender, by exploring whether employee welfare has a different supportive role on work-to-life and life-to-work integration and mental health as a function of employees’ gender.

### The current study

This Study investigates the impact of employee welfare on employees’ mental health, with a focus on gender differences on a large scale of Italian employees. Specifically, we examined whether the relationship between employee welfare and mental health is mediated by reduced perceptions of work-interference-private-life (WIPL) and private-life-interference-work conflict (PLIW).

We tested the following hypotheses:

H1: Women report lower levels of mental health than men, even after accounting for relevant sociodemographic characteristics (i.e., age, workplace role, relationship status, and parenthood).

H2: Employee welfare is positively associated with mental health through reduced perceptions of both WIPL and PLIW.

Additionally, we explored whether gender moderates this mediational relationship, assessing if the effect of welfare on mental health through reduced WIPL and PLIW differs between men and women.

## Methods

### Data collection and participants

The data were collected from four large Italian companies in the following sectors: transport, insurance, telecommunications, and large-scale retail trade of food and consumer goods. Participants were contacted by email through the companies’ Human Resources Offices between September 2020 and July 2024. The email was distributed to all employees by the HR office in each of the four companies. The email presented the study and contained a link to an anonymous online survey. The online survey was created and administered using the Qualtrics Platform. The overall response rate was 38%. The demographic composition of the sample, particularly with respect to gender and age, closely matched that of the workforce in the participating organizations, supporting the representativeness of the sample on these characteristics.

The research was carried out following The Code of Ethics of the World Medical Association (Declaration of Helsinki) for research involving humans. It was designed to inform the companies’ human resources offices about the impact of welfare policies among workers to improve their quality. Data were related to the employee welfare surveillance activity, which does not require approval by an ethical committee or to obtain informed consent from participants. All participants volunteered for the study.

A sample of 7180 employees participated in the online survey, equally divided into males (51.5%) and females (48.5%). Most respondents were between 41 and 50 (35.0%) or over 50 (35.7%). Most respondents were educated to high school diploma level (52.1%) or university (45.0%). Participants self-reported their employment status through the classification of workers under the Italian labour law (Art. 2095, Codice Civile Italiano), which differentiates levels of job status (usually corresponding to decreasing levels of income and time flexibility). Response options were managers (1.8%), supervisors (20.6%), and clerks (77.6%). Employees who performed roles specific to the nature of each company and who could not be classified in the previous roles were not included in the analyses due to the limited overlap between companies in different sectors. Most of the sample had a partner at the time of the survey (74.9%); almost half (43.6%) were people with children. Table 1 summarizes the sociodemographic characteristics of the participants.

**Table 1:** Sociodemographic characteristics of participants (n = 7180)

Sociodemographic Characteristics	*n*	%
Gender		
Female	3484	48.5
Male	3696	51.5
Age		
Under 30	395	5.5
31–40	1708	23.8
41–50	2512	35.0
Over 50	2557	35.7
Highest educational level		
Middle school	205	2.9
High school	3733	52.1
University or postgraduate degree	3224	45.0
Workplace role		
Manager	132	1.8
Supervisor	1475	20.6
Clerk	5573	77.6
Relationship		
Single	1793	25.1
In couple	5359	74.9
Parenthood		
Without children	4043	56.4
With children	3123	43.6

### Measures

#### Mental health

This variable was measured through the World Health Organization-Five Well-Being Index (WHO-5), Italian translation [11]. Responses were based on a 6-point Likert scale ranging from 0 (at no time) to 5 (all the time), with higher total scores indicating greater mental health. An example item is “I have felt cheerful and in good spirits”. The raw score is calculated by adding the numbers of the five responses. The raw score ranges from 0 to 25, where 0 represents the worst possible health, and 25 represents the best. A score below 13 indicates a poor state of health. Previous studies have documented good reliability and validity across different populations [12]. According to Cronbach [44], the scale’s internal consistency in the present sample was good (Cronbach’s alpha = 0.89).

### Satisfaction with employee welfare

A single item was used to measure satisfaction with employee welfare: “Overall, how satisfied are you with the welfare initiatives implemented by your company?” Participants were asked to evaluate it on a scale from 0 (completely dissatisfied) to 5 (completely satisfied). The use of a single-item measure was considered appropriate because the construct was conceptualized as an overall evaluative judgment of organizational welfare provisions rather than a multidimensional construct. Previous methodological research has shown that single-item measures can provide valid assessments of global attitudes and satisfaction constructs when the underlying concept is concrete and unidimensional [45].

### Work-life conflict

This variable was measured through an adapted version of the Quality of Work-Life Scale [46, 47], reported in the Supplemental Material. The original scale was a 16-item measure designed to assess four work-life spillover aspects: positive and negative work-to-family spillover, positive and negative family-to-work spillover. Items are rated on a 5-point Likert scale (ranging from 1 = never to 5 = all of the time), with higher total scores indicating more significant levels of each specific aspect of work-life spillover. For the current study, a shorter, 6-item version of this scale was used (Work Interference with Private Life, or WIPL: 3 items; Private Life Interference with Work, or PLIW: 3 items). The shortened version was developed by selecting the items with the highest conceptual relevance for each dimension (WIPL and PLIW) from the original instrument. Items were translated and adapted to the Italian context following a translation and review procedure conducted by the research team. Examples are “Job stress makes me irritable at home (negative work-to-life spillover)” and “Personal worries distract me on the job (negative life-to-work spillover)”. Because we used a brief Italian version of this scale not previously validated, we performed Confirmatory Factor Analysis (CFA) to test the scale’s validity. The results indicated that the two-factor solution fit the data adequately (Chi-squared = 204, df = 8, *p* < 0.001; CFI = 0.989; SRMR = 0.023; RMSEA = 0.054). All standardized factor loadings were statistically significant and ranged from 0.56 to 0.90 for WIPL and from 0.70 to 0.85 for PLIW. The scale’s internal consistency in the present sample was good for both the WIPL (Cronbach’s alpha = 0.79) and PLIW (Cronbach’s alpha = 0.80) subscales.

### Data analysis

Analyses were performed using Jamovi (Version 2.3.28, The Jamovi project, 2022, retrieved from https://www.jamovi.org) and IBM SPSS Statistics, version 29 (SPSS, Chicago, IL, USA).

Descriptive statistics were calculated on the sample’s sociodemographic and psychological characteristics. Mean and standard deviation (SD) were reported for continuous variables, and percentages were reported for categorical variables. The normal distribution of the data was tested by calculating skewness and kurtosis indices; the recommended range of ± 2 and ± 7 was considered for normality, respectively [48].

Confirmatory Factor Analysis (CFA) was performed to test the validity of the Work-Life Conflict scale, adapted for the present study. Hu and Bentler’s guidelines [49] were used to determine if the expected model was plausible based on the data. Parameter estimates were computed using a maximum likelihood estimation method, while an optimal model fit was evaluated using the following criteria: a comparative fit index (CFI) of 0.95 or more, a standardized root mean squared residual (SRMR) of 0.05 or less, and a root mean square error of approximation (RMSEA) of 0.05 or less. Cronbach’s α was calculated to examine internal consistency. Cronbach’s α higher than 0.60 was considered acceptable [50].

A linear mixed model with restricted maximum likelihood (REML) estimation (Satterthwaite method for degrees of freedom) as implemented in Jamovi [51] was performed to describe in terms of sociodemographic profile the Mental Health of workers. Mental Health was the dependent variable. Gender was the primary predictor of interest, consistent with H1. Age (2 levels: under 50 vs. over 50), workplace role (3 levels: manager, supervisor, and clerk), relationship status (2 levels: single vs. in couple), and parenthood (2 levels: without children vs. with children) were included in the model to account for sociodemographic characteristics that have been previously associated with employees’ psychological well-being and work-related experiences. Including these variables allowed us to estimate gender differences in mental health while controlling for potential confounding factors.

**Fig. 1:**
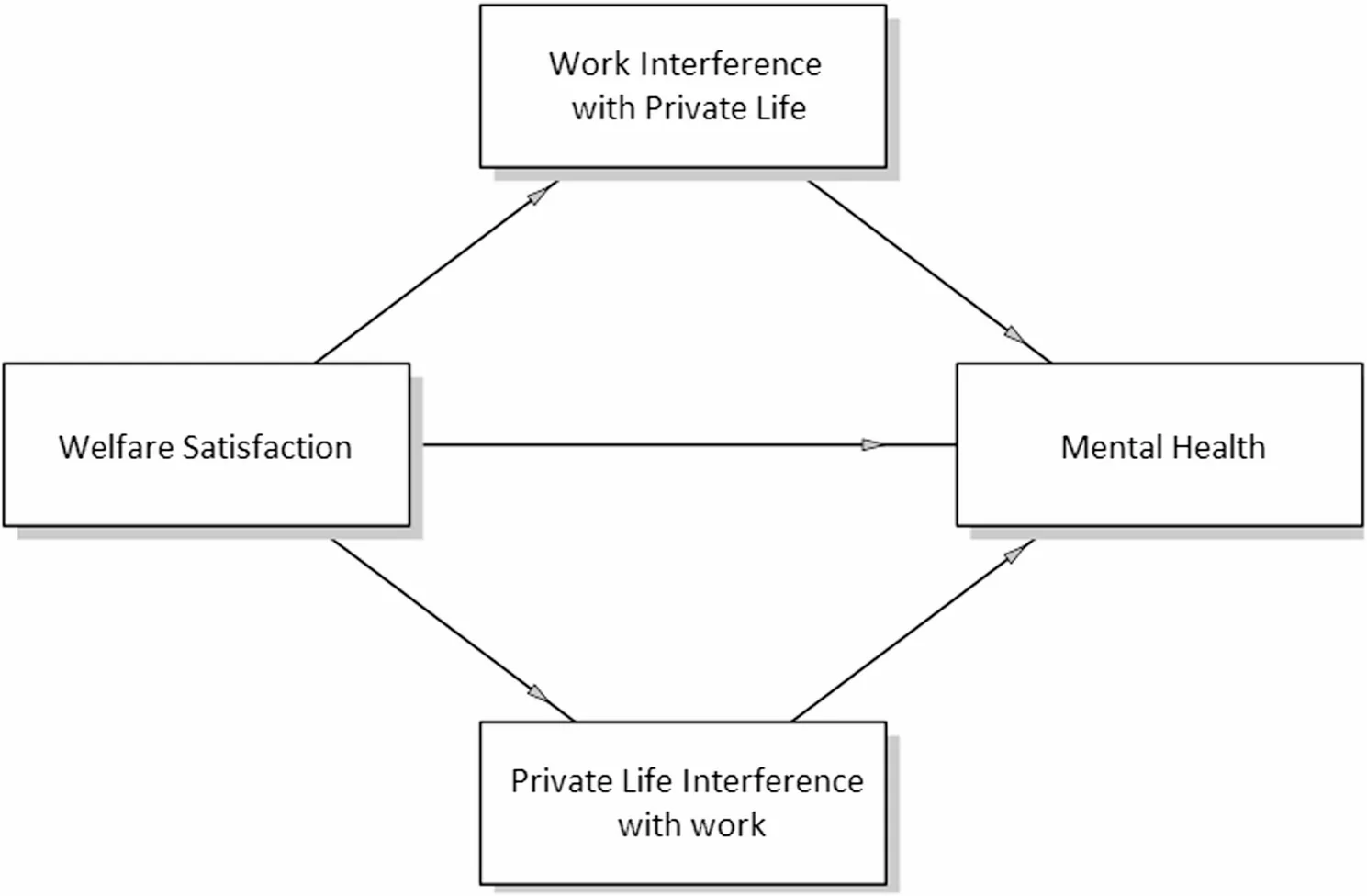
Conceptual model linking employee welfare, work-life conflict and mental health

Two moderated mediation models were performed to investigate employee welfare’s role in reducing work-life conflict and enhancing mental health for both men and women. Mainly, the GLM Mediation Model in the MedMod module for data analysis in Jamovi was used to analyse the mediation models for Mental Health; the method was based on the mediation method with 5000 bootstrap bias-corrected 95% confidence intervals, recommended by Hayes [52] as measures of indirect effects. Two mediation models were constructed in this study: in the first model, the two subscales of work-life conflict (WIPL and PLIW) were selected as the mediator variables, satisfaction with the employee welfare was the independent variable, and the total score of WHO-5 was the dependent variable; in the second model, the moderator effect of gender was tested. Figure 1 illustrates the conceptual model.

Effect sizes for the linear mixed model and the moderated mediation models were reported as standardized regression coefficients (β). In addition, model fit was evaluated using marginal and conditional R² for mixed-effects models.

## Results

The results are organized as follows. We begin by presenting descriptive statistics for mental health, satisfaction with employee welfare, and work–life conflict in the final analytic sample (*N* = 7180; 38% response rate). We then use a linear mixed model to describe mental health differences across sociodemographic characteristics. Finally, we report the findings from the moderated mediation analyses testing whether employee welfare is associated with mental health through reduced work-to-life and life-to-work conflict, and whether these indirect pathways differ for men and women.

Descriptive statistics for all variables of interest are reported in (Table 2). All variables were normally distributed. The WHO-5 total score was below the threshold of 13 in 2921 workers (40.7% of the sample), suggesting poor Mental Health in a significant portion of workers.

**Table 2:** Descriptive statistics of the scales used in the study

	WHO-5	WIPL	PLIW	Welfare Satisfaction
*N*	7180	7154	7150	7007
Mean	13.60	2.48	1.49	3.30
SD	5.43	0.91	0.58	0.83
Minimum	0	1	1	1
Maximum	25	5	5	5
Skewness	-0.22	0.68	1.93	-0.07
SE skewness	0.03	0.03	0.03	0.03
Kurtosis	-0.67	-0.14	5.84	0.03
SE kurtosis	0.06	0.06	0.06	0.06

*WHO-5* Mental health, *WIPL *Work Interference with Private Life, *PLIW* Private Life Interference with Work, Welfare Satisfaction Satisfaction with the employee welfare, *SD *Standard Deviation, *SE *Standard Error

The LMM successfully converged and explained 3% of the variance (Rc^2^=0.03). The fixed effects explained the 2% alone (Rm^2^=0.02). Fixed effects are summarized in (Table 3).

**Table 3:** Linear mixed model of mental health. Fixed effects omnibus tests

Effect	F	Numerator df	Denominator df	*p*
Gender	83.35	1	6933	< 0.001
Relationship	13.35	1	7129	< 0.001
Parenthood	2.34	1	7019	0.126
Workplace role	8.19	2	7124	< 0.001
Age	0.74	1	7035	0.390

Number of Observations: 7138; df = degrees of freedom

**Fig. 2:**
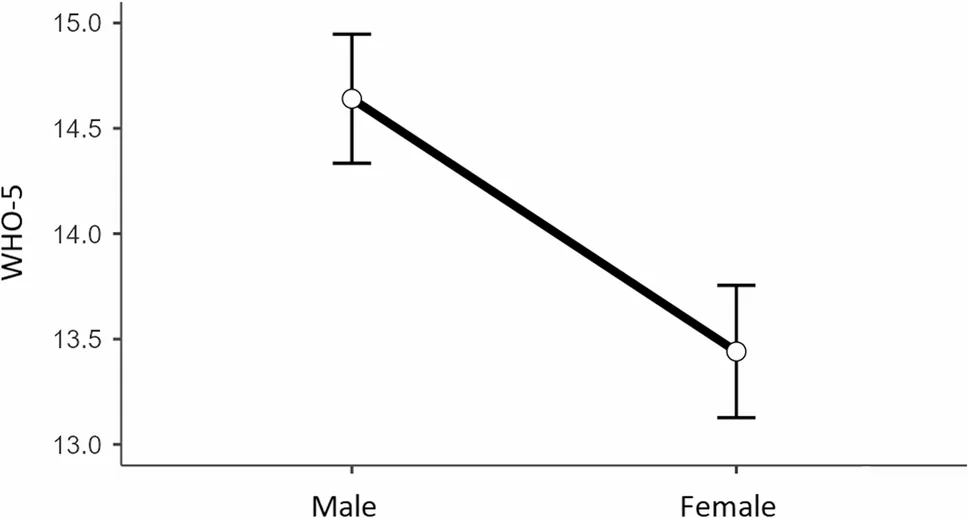
Marginal means of mental health in function of gender. Note: The vertical bars represent standard errors

The factor ‘Gender’ was significant (F1,6933 = 83.35, *p* < 0.001; β = 0.22). Men reported a higher mental health (Mean = 14.6; SE = 0.31) than women (Mean = 13.4; SE = 0.31; see Fig. 2). 

The factor ‘Relationship’ was also significant (F1,7129 = 13.35, *p* < 0.001; β = 0.10). Workers in couples reported a higher mental health (Mean = 14.6; SE = 0.30) than singles (Mean = 13.8; SE = 0.32).

**Fig. 3:**
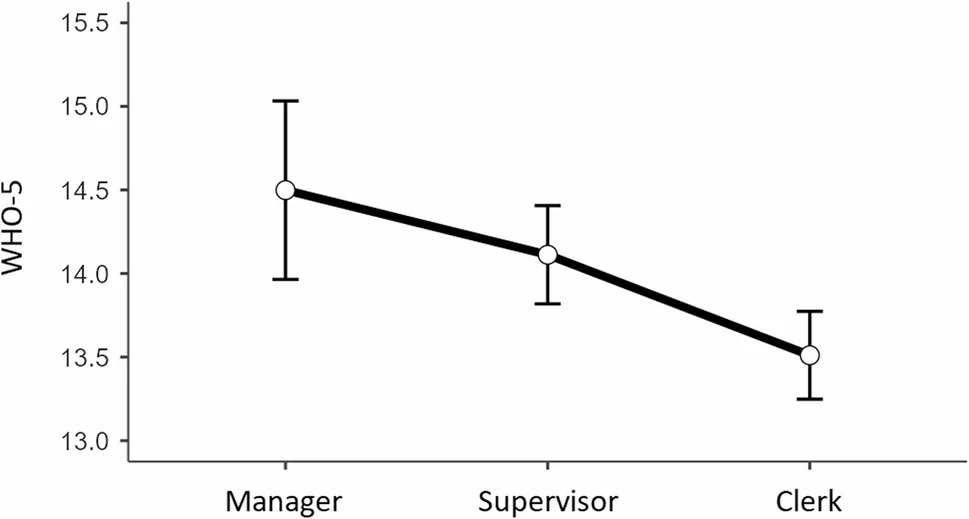
Marginal means of mental health in function of the workplace role

Finally, the factor ‘Workplace role’ was significant (F2,7124 = 8.19, *p* < 0.001). The effect was accounted for by a significant difference between clerks and supervisors (post hoc comparisons with Bonferroni’s correction evidenced a p-value < 0.001; β = 0.18). In contrast, no statistically significant difference between managers and supervisors was observed. Indeed, Mental Health was similar between managers (Mean = 14.5; SE = 0.53) and supervisors (Mean = 14.1; SE = 0.29), whereas the Mental Health of clerks was poorer (Mean = 13.5; SE = 0.26; see Fig. 3). 

The likelihood ratio test (LRT) indicated that the random intercept for the company improved the model (LRT = 20.60; *p* < 0.001). The ICC was very low (0.008), thus suggesting that it was not necessary to consider the company’s random effect in subsequent analyses.

The first moderated mediation model suggested that all direct and indirect effects were significant (Table 4). Regarding the direct effects, results suggested that higher satisfaction with welfare was significantly associated with lower work-to-life conflict (*p* < 0.001) and lower life-to-work conflict (*p* < 0.05). Moreover, lower work-to-life and life-to-work conflicts were associated with higher mental health (*p* < 0.001). Regarding the indirect effects, results evidenced that higher satisfaction with welfare was associated with higher mental health through a mediating effect of both work-to-life conflicts (*p* < 0.001) and life-to-work conflict (*p* < 0.05).

**Table 4:** Standardized regression weights for the moderated mediation model linking employee welfare, work-life conflict and mental health

				95% CI				
Type	Effect	b	SE	Lower	Upper	β	z	*p*
Indirect	Welfare Satisfaction ⇒ WIPL ⇒ WHO-5	0.1717	0.01843	0.13558	0.20785	0.02618	9.32	< 0.001
	Welfare Satisfaction ⇒ PLIW ⇒ WHO-5	0.0197	0.00889	0.00230	0.03716	0.00301	2.22	0.027
Component	Welfare Satisfaction ⇒ WIPL	-0.1478	0.01310	-0.17350	-0.12214	-0.13380	-11.28	< 0.001
	WIPL ⇒ WHO-5	-1.1616	0.07032	-1.29948	-1.02382	-0.19568	-16.52	< 0.001
	Welfare Satisfaction ⇒ PLIW	-0.0193	0.00846	-0.03590	-0.00274	-0.02733	-2.28	0.022
	PLIW ⇒ WHO-5	-1.0211	0.10897	-1.23465	-0.80751	-0.11005	-9.37	< 0.001
Direct	Welfare Satisfaction ⇒ WHO-5	1.2555	0.07461	1.10928	1.40176	0.19144	16.83	< 0.001
Total	Welfare Satisfaction ⇒ WHO-5	1.4470	0.07642	1.29718	1.59675	0.22063	18.93	< 0.001

*b  *unstandardized coefficient, *SE* standard error, *CI*  confidence interval (95%), *β*  standardized coefficient

The second moderated mediation model confirmed the results of the first model. Indeed, satisfaction with welfare was associated with lower work-to-life conflict and with lower life-to- work conflict, leading to improved mental health for both genders (Table 5).

**Table 5:** Standardized regression weights for the moderated mediation model linking gender, employee welfare, work-life conflict and mental health

					95% CI				
Gender	Type	Effect	b	SE	Lower	Upper	β	z	*p*
Average	Indirect	Welfare Satisfaction ⇒ WIPL ⇒ WHO-5	0.1730	0.01851	0.13674	0.20929	0.02645	9.35	< 0.001
Average		Welfare Satisfaction ⇒ PLIW ⇒ WHO-5	0.0201	0.00924	0.00195	0.03818	0.00307	2.17	0.030
Average	Component	Welfare Satisfaction ⇒ WIPL	-0.1461	0.01308	-0.17175	-0.12046	-0.13236	-11.17	< 0.001
Average		WIPL ⇒ WHO-5	-1.1842	0.06925	-1.31993	-1.04847	-0.19986	-17.10	< 0.001
Average		Welfare Satisfaction ⇒ PLIW	-0.0188	0.00845	-0.03535	-0.00224	-0.02662	-2.23	0.026
Average		PLIW ⇒ WHO-5	-1.0674	0.10822	-1.27950	-0.85529	-0.11523	-9.86	< 0.001
Average	Direct	Welfare Satisfaction ⇒ WHO-5	1.2459	0.07369	1.10150	1.39034	0.19050	16.91	< 0.001
Average	Total	Welfare Satisfaction ⇒ WHO-5	1.4485	0.07579	1.29996	1.59704	0.22087	19.11	< 0.001
male	Indirect	Welfare Satisfaction ⇒ WIPL ⇒ WHO-5	0.2295	0.02955	0.17157	0.28740	0.03499	7.77	< 0.001
male		Welfare Satisfaction ⇒ PLIW ⇒ WHO-5	0.0177	0.01070	-0.00324	0.03869	0.00270	1.66	0.098
male	Component	Welfare Satisfaction ⇒ WIPL	-0.1725	0.01826	-0.20833	-0.13675	-0.15631	-9.45	< 0.001
male		WIPL ⇒ WHO-5	-1.3301	0.09752	-1.52122	-1.13893	-0.22386	-13.64	< 0.001
male		Welfare Satisfaction ⇒ PLIW	-0.0203	0.01179	-0.04340	0.00281	-0.02875	-1.72	0.085
male		PLIW ⇒ WHO-5	-0.8732	0.14323	-1.15394	-0.59248	-0.09401	-6.10	< 0.001
male	Direct	Welfare Satisfaction ⇒ WHO-5	1.2180	0.10318	1.01578	1.42025	0.18572	11.80	< 0.001
male	Total	Welfare Satisfaction ⇒ WHO-5	1.4760	0.10563	1.26900	1.68307	0.22506	13.97	< 0.001
female	Indirect	Welfare Satisfaction ⇒ WIPL ⇒ WHO-5	0.1243	0.02275	0.07968	0.16883	0.01902	5.46	< 0.001
female		Welfare Satisfaction ⇒ PLIW ⇒ WHO-5	0.0218	0.01552	-0.00860	0.05225	0.00334	1.41	0.160
female	Component	Welfare Satisfaction ⇒ WIPL	-0.1197	0.01874	-0.15641	-0.08293	-0.10841	-6.38	< 0.001
female		WIPL ⇒ WHO-5	-1.0383	0.09835	-1.23110	-0.84557	-0.17547	-10.56	< 0.001
female		Welfare Satisfaction ⇒ PLIW	-0.0173	0.01210	-0.04102	0.00642	-0.02450	-1.43	0.153
female		PLIW ⇒ WHO-5	-1.2616	0.16226	-1.57962	-0.94355	-0.13637	-7.77	< 0.001
female	Direct	Welfare Satisfaction ⇒ WHO-5	1.2738	0.10522	1.06759	1.48005	0.19502	12.11	< 0.001
female	Total	Welfare Satisfaction ⇒ WHO-5	1.4210	0.10871	1.20791	1.63403	0.21667	13.07	< 0.001

 Gender moderated the indirect effect of employee welfare on mental health through work-to-life conflict, but this pattern was specific to the WIPL pathway. The indirect effect via WIPL was stronger for men than for women (Table 6). Consistently, the interaction term between gender and WIPL in predicting mental health was statistically significant (*p* = 0.035), supporting the presence of moderated mediation for this pathway. In contrast, no significant moderated mediation was found for the PLIW pathway. The interaction term between gender and PLIW in predicting mental health was not statistically significant (*p* = 0.073), indicating that the indirect effect via PLIW does not vary as a function of gender.

**Table 6:** Moderation effects of the moderated mediation model linking gender, employee welfare, work-life conflict and mental health

				95% CI				
Moderator	Interaction	b	SE	Lower	Upper	β	z	*p*
	Welfare Satisfaction: Gender ⇒ WIPL	0.05287	0.0262	0.00158	0.1042	0.02395	2.020	0.043
	Welfare Satisfaction: Gender ⇒ PLIW	0.00300	0.0169	-0.03011	0.0361	0.00212	0.177	0.859
	Welfare Satisfaction: Gender ⇒ WHO-5	0.05580	0.1474	-0.23304	0.3446	0.00427	0.379	0.705
	Gender: WIPL ⇒ WHO-5	0.29174	0.1385	0.02027	0.5632	0.07129	2.106	0.035
	Gender: PLIW ⇒ WHO-5	-0.38837	0.2164	-0.81258	0.0358	-0.05761	-1.794	0.073

The index of moderated mediation for the WIPL pathway is significantly different from zero, as indicated by a confidence interval that does not include zero, supporting the presence of moderated mediation. In contrast, the index for the PLIW pathway includes zero, indicating no evidence of moderated mediation

## Discussion

The COVID-19 pandemic has underscored gender disparities in mental health, with women experiencing more pronounced declines in mental health [15], often linked to higher levels of work-life conflict than those faced by men [53]. Within this context, employee welfare programs may show potential as strategies to assist employees in managing work and personal life demands [43, 42]. This study explored the role of employee welfare in bolstering mental health through the alleviation of work-life conflict in a substantial sample of Italian employees, with a specific focus on examining gender differences.

Preliminary results reveal that women reported lower mental health levels compared to men, a finding that aligns both with our hypothesis (H1) and with a substantial body of literature indicating a consistent gender gap in mental health outcomes [17, 16]. This gender disparity suggests that women may face unique stressors or barriers to mental health, potentially due to societal expectations or differing role demands [54]. Furthermore, clerks reported significantly lower mental health levels compared to individuals in supervisory or managerial roles, a result that corroborates earlier research linking lower occupational status with an elevated risk of mental health issues [55, 56]. This association may reflect the increased stress and limited resources available to individuals in lower-status positions. Lastly, individuals in couples reported higher levels of mental health than those that are single, hinting that family roles may contribute to a sense of psychological stability or well-being. This result aligns with previous findings suggesting that family commitment can provide individuals with additional sources of social support and purpose, which may positively impact mental health [57].

Regarding our second hypothesis (H2), our findings highlight the beneficial role of employee welfare programs in alleviating work-life conflict and supporting mental health. While these programs effectively reduced both work-to-life and life-to-work conflicts and enhanced mental health, their influence was notably stronger on work-to-life conflict than on life-to-work conflict. The limited effect of employee welfare programs on life-to-work conflict may arise from employees’ perceptions that they bear personal responsibility for managing challenges in their private lives. Research suggests that some individuals may feel a stronger sense of control and ownership over personal matters, such as family caregiving or health issues, compared to work-related responsibilities [58]. This self-reliance is reinforced by societal norms and expectations that encourage individuals to independently handle personal matters without seeking external help. For instance, in recent years, intensive parenthood has become a dominant approach to parenting, where parents focus much of their time and energy on meeting their children’s needs. This approach often leads to a sense of social obligation, as parents feel pressured to align with societal expectations and uphold the norm of full-time caregiving [59]. Consequently, many parents take on the responsibility of managing family duties independently, despite the potential for conflict with work demands. This phenomenon reflects broader cultural norms that promote intensive caregiving as the ideal, which can limit parents’ willingness to seek external support, including from workplace welfare programs. As a result, welfare programs may have a reduced effect on life-to-work conflict, especially when personal and family responsibilities are internalized as private duties to be handled alone.

Our findings can also be read in light of established evidence on gender differences in the two directions of work–life conflict. Prior research consistently shows that women more often report work-to-family interference than men, whereas gender gaps in family-to-work interference tend to be smaller or context-dependent [7, 8, 24]. Systematic reviews likewise document that women carry a disproportionate share of unpaid domestic and care work, which amplifies work–life strain [16, 28]. Longitudinal work further indicates that work-to-family and family-to-work conflict follow partially distinct pathways and may not respond to the same organizational levers [21]. Considered together, these findings help explain why employee welfare in our sample was more closely linked to reduced work-to-life conflict, and why gender moderated this pathway, while associations involving life-to-work conflict were weaker and not significantly moderated by gender.

Importantly, gender moderated the indirect association between employee welfare and mental health through work-to-life conflict, such that the benefits of welfare were more pronounced for men than for women (Table 6). By contrast, the interaction involving life-to-work conflict did not reach statistical significance, indicating that the gendered pattern observed here is primarily confined to the work-to-life pathway rather than to spillover from private life into work. Because our design did not assess welfare uptake, delegation of caregiving, or employees’ reasons for using (or not using) organizational provisions, any explanation of why welfare appears less effective for women along this pathway must remain tentative. Drawing on prior literature, one post hoc interpretation is that employee welfare may translate less readily into reduced work-to-life conflict among women when family-side demands remain largely self-managed. In the Italian context, where women continue to perform the majority of unpaid domestic and care work [25], organizational initiatives oriented toward work scheduling or workplace support may do little to alleviate conflict that originates in the private sphere. Consistent with this possibility, previous studies suggest that women are often less willing than men to delegate caregiving tasks, as delegation may feel incongruent with stereotypical gender roles and may elicit guilt when outside help is used [60, 61]. Cultural ideals of intensive parenting may further reinforce the view that family care is a personal responsibility rather than a domain legitimately supported by employers [59]. We therefore present these arguments as theoretically informed hypotheses, not as mechanisms tested in the present study. Future research should examine whether gendered patterns of welfare use, caregiving load, and delegation help explain for whom, and through which pathway, employee welfare translates into improved mental health.

From a managerial perspective, supervisors are often the first organizational contact through whom employees experience support, or constraint, in balancing work and family demands. Training managers to adopt family-supportive supervisor behaviours, including emotional support, instrumental assistance, role modelling, and creative work–family management, may help them recognize gendered patterns of conflict and respond appropriately, particularly when family obligations spill over into work [62]. Intervention research shows that such training can improve job satisfaction and physical health among employees with high family-to-work conflict [62]. Line managers should also be encouraged to normalize benefit uptake and to monitor whether welfare resources are accessed equitably across genders, as organizational support is most effective when employees perceive genuine backing from immediate supervisors [42].

These findings underscore the constraints of employee welfare programs in contexts where organizational and societal climates may lack comprehensive support for reducing gender disparities. Research suggests that HR practices are most effective when embedded within an organizational culture that is genuinely perceived as supportive [42]. In many cases, organizations may introduce welfare initiatives mainly for reputational purposes [35], potentially resulting in employees perceiving these initiatives as inauthentic. In such cases, employees may hesitate to engage with welfare resources due to concerns about judgment or a perceived lack of genuine support.

For HR professionals, our results imply that welfare portfolios should be designed to address both directions of work–life conflict rather than focusing predominantly on work-to-life spillover. Because life-to-work conflict was less responsive to welfare satisfaction in our sample, HR policies should explicitly include provisions targeting the private sphere, such as subsidized childcare or eldercare, paid caregiver leave, and domestic workload supports, alongside flexible scheduling arrangements [36, 34]. Flexible credit or cafeteria-style benefit plans, which allow employees to select from a menu of options aligned with their needs, represent one mechanism for delivering tailored support; prior evaluations link flexible credit benefit plans to improved employee health outcomes [63], and meta-analytic evidence on corporate well-being programs highlights the value of tailoring benefits to employee characteristics, including gender [64]. Employee needs assessments, including focus groups and surveys analysed by gender and caregiving status, can inform menu design and ensure that benefits address life-to-work as well as work-to-life strain [65]. Overall, these results suggest that employee welfare programs contribute positively to mental health by mitigating work–life conflict, but generic packages may not suffice to close the gender gap. Combining tailored, flexible benefit designs with manager training and HR policies that explicitly recognize life-to-work conflict may offer a more equitable path toward supporting workers’ mental health.

Beyond organizational actors, policymakers have a role in shaping the conditions under which firm-level welfare can reduce gendered mental health inequalities. Because life-to-work conflict in our data proved less responsive to welfare satisfaction, interventions confined to the workplace may leave intact the structural drivers of women’s mental burden, particularly the unequal distribution of unpaid care [16, 25, 29]. Public policies that expand affordable and accessible childcare and eldercare, support paid leave for caregivers, and incentivize employers to offer flexible, family-oriented benefit packages could complement corporate welfare and extend its reach to employees whose strain originates outside the workplace [34, 36, 38]. Monitoring uptake and mental health outcomes by gender within national and sectoral frameworks would also help evaluate whether work–life policies equitably benefit those who face the greatest conflict, rather than primarily employees whose demands originate at work.

### Limitations and future directions

Several limitations warrant consideration in interpreting these findings. Firstly, the reliance on self-reported data introduces potential bias due to social desirability. Secondly, because participation was voluntary, the possibility of non-response bias cannot be entirely excluded. Employees who chose to participate may have differed from non-participants in their perceptions of organizational welfare, work-life conflict, or psychological well-being. Therefore, caution is warranted when generalizing the findings beyond the participating organizations. Additionally, this study did not assess organizational climate or culture, factors that could help determine whether welfare practices are genuinely intended to support employee well-being or are primarily reputational in nature [35, 42]. Future research could incorporate organizational climate measures to assess its role in shaping the effectiveness of welfare initiatives. Furthermore, the cross-sectional design limits causal inference: we cannot establish the temporal order of associations among welfare satisfaction, work–life conflict, and mental health, and reverse causality cannot be ruled out (for example, employees with poorer mental health may perceive welfare provisions less favourably or report greater conflict). As in other cross-sectional examinations of work–life conflict and well-being [22, 23], our estimates therefore describe contemporaneous associations rather than confirmed causal effects. Longitudinal and quasi-experimental designs are needed to clarify directionality, examine whether welfare effects persist over time, and assess whether changes in organizational provisions precede improvements in mental health.

## Conclusion

Research consistently highlights a gender gap in mental health, often linked to heightened work-life conflict among women [1, 7]. Employee welfare programs present a valuable opportunity to alleviate perceived work-life conflict and enhance well-being. This study demonstrates that welfare programs effectively reduce work-to-life conflict and, with minor strength, life-to-work conflict, however, their impact is more limited for women than men. These findings point to the need for more targeted welfare programs tailored to address the specific needs of diverse employee groups. Tailored welfare programs that directly address the unique challenges faced by women in balancing work and personal life demands could better support mental health across the workforce, ultimately fostering a more inclusive and supportive workplace environment.

## Supplementary Information

Supplementary Material 1.

## Data Availability

The data that support the findings of this study are available from Catholic University of the Sacred Heart, but restrictions apply to the availability of these data, which were used under license for the current study and so are not publicly available. The data are, however, available from the authors upon reasonable request and with the permission of Catholic University of the Sacred Heart.

## Abbreviations

WIPL: Work-interference-with-private-life
PLIW: Private-life-interference-with-work
WHO-5: World Health Organization-Five Well-Being Index
HR: Human resources
LMM: Linear mixed model
CFA: Confirmatory factor analysis
CFI: Comparative fit index
SRMR: Standardized root mean squared residual
RMSEA: Root mean square error of approximation
SD: Standard deviation
SE: Standard error
CI: Confidence interval
