# Supplementary material for: Balancing work and life: how employee welfare programs influence mental health with a gendered lens
Source: BMC Psychol. 2026 Jul 24;14:1510. doi: 10.1186/s40359-026-05235-5 (PMC13682162; doi:10.1186/s40359-026-05235-5)
Supplement: Supplementary file 1 [file 40359_2026_5235_MOESM1_ESM.docx]

**Supplemental Material**

**Adapted Quality of Work-Life Scale**

1. My workload reduces my engagement in my personal life activities (negative work-to-life spillover)
2. Job stress makes me irritable at home (negative work-to-life spillover)
3. Work problems distract me in my personal life (negative work-to-life spillover)
4. The responsibilities I have in my personal life reduce the effort I put into work (negative life-to-work spillover)
5. Personal worries distract me on the job (negative life-to-work spillover)
6. Stress in my personal life makes me irritable at work (negative life-to-work spillover)
